# Use of Vitamin C as Placebo in Anesthesiology

**DOI:** 10.5812/aapm.8055

**Published:** 2013-01-01

**Authors:** Masood Mohseni

**Affiliations:** 1 Department of Anesthesiology, Rasoul Akram Medical Center, Iran University of Medical Sciences (IUMS), Tehran, Iran

**Keywords:** Placebos, Clinical Trial, Anesthesia, Pain

Dear Editor,

I was interested to read the paper entitled "Effects of clonidine premedication upon postoperative shivering and recovery time in opium addicted and non-addicted patients by elective leg fracture surgeries" (1). I note that the authors administered vitamin C tablet as placebo for clonidine. Noteworthy, earlier animal studies have suggested that there is a close relationship between recovery from anesthesia and extracellular ascorbic acid levels (2). Vitamin C has showed pain relieving effects (3) namely in complex regional pain syndrome (4), post herpetic neuralgia (5), migraine headache (6) and myalgia associated with the administration of suxamethonium (7). Moreover, the placebo tablet should be identical-looking with similar color, taste, and smell with the treatment drug. I guess that vitamin C with its particular taste and smell could be easily distinguished from clonidine. Taken together, I believe that since vitamin C has the potential to affect outcome in this study, it may not be an appropriate choice for placebo.
